# Second Law Analysis of Spectral Radiative Transfer and Calculation in One-Dimensional Furnace Cases

**DOI:** 10.3390/e21050461

**Published:** 2019-05-02

**Authors:** Shiquan Shan, Zhijun Zhou

**Affiliations:** State Key Laboratory of Clean Energy Utilization, Zhejiang University, Hangzhou 310027, China

**Keywords:** thermodynamics second law, exergy, entropy, spectral radiation transfer

## Abstract

This study combines the radiation transfer process with the thermodynamic second law to achieve more accurate results for the energy quality and its variability in the spectral radiation transfer process. First, the core ideas of the monochromatic photon exergy theory based on the equivalent temperature and the infinite-staged Carnot model are reviewed and discussed. Next, this theory is combined with the radiation transfer equation and thus the spectral radiative entropy and the radiative exergy transfer equations are established and verified based on the second law of thermodynamics. Finally, one-dimensional furnace case calculations are performed to determine the applicability to engineering applications. It is found that the distribution and variability of the spectral radiative exergy flux in the radiation transfer process can be obtained using numerical calculations and the scatter media could slightly improve the proportion of short-wavelength radiative exergy during the radiation transfer process. This has application value for research on flame energy spectrum-splitting conversion systems.

## 1. Introduction

Thermal radiation is the main energy transfer method in high-temperature energy conversion systems; it plays an important role in combustion boilers, solar energy systems, and aerospace power equipment [1]. The analysis of the thermal radiation process using the second law of thermodynamics has unique significance. On the one hand, radiation is important in high-temperature conditions. The second law analysis of the radiation heat transfer process allows for the estimation of the entropy generation and exergy loss in furnaces [2] or high-temperature equipment [3] so that more reasonable evaluations and optimizations can be conducted for energy conversion systems. On the other hand, in order to achieve an effective utilization of high-temperature radiative energy in combustion energy conversion system, radiative energy spectrum-splitting conversion [4] in combustion progress is required [5]; that is, radiative energy from combustion flame is separated [6,7], the short-wavelength radiation could be used for photovoltaic conversion, and the remaining thermal radiation is converted through traditional thermal power cycles, this constitutes to a combustion flame grading power generation system [5]. Thus, this requires the thermodynamic second law for an accurate analysis of the spectral exergy in radiation transfer processes; it further guides the energy management with regard to quality and achieves exergy efficient conversion. Therefore, second law analysis of spectral radiative transfer is very important for the combustion flame grading power generation system.

To date, scholars have conducted research on the radiative thermodynamics and the application of the second law in radiation transfer calculations. The following sections introduce these two topics and make some comments.

### 1.1. Radiative Thermodynamics

Radiation is an electromagnetic wave, which possesses the characteristics of wavelength and frequency; as a means of heat transfer, however, radiation has entropy and exergy characteristics similar to thermal energy. It is evident that radiative energy has unique characteristics; therefore, there is a certain difference between radiative energy and thermal energy. Wright [8] investigated the entropy of radiative heat transfer and the entropy of the heat conduction process and described their differences. Liu and Chu [9] examined the expression of entropy generation in heat transfer and demonstrated that the traditional equation for the entropy generation rate of conduction is not suitable for the local entropy generation rate of the radiative heat transfer.

There are various equations for the exergy of blackbody radiation [10,11,12] and these have been discussed by Bejan [13]. At present, scholars recognize and accept the exergy equation of blackbody radiation proposed by Petela [10]; many researchers amended or expanded this equation [14,15,16,17,18] and it was also used in solar energy system analysis [19,20,21]. Recently, we discussed different equations and explained the rationality of Petela’s equation by establishing a radiation machine model [22]. For the spectral radiative exergy, Candau [23] performed a derivation of the spectral exergy intensity based on Planck’s equation of spectral radiative entropy intensity and the radiation temperature concept; however, this study is also designed for the whole total spectrum radiation analysis. Chen et al. [24,25] proposed a corresponding expression of spectral radiative exergy based on the concept of equivalent temperature; however, these studies treated radiative energy as thermal energy. In order to analyze spectrum-splitting energy conversion system appropriately, there needs to be a spectral exergy theory based on the ability of a monochromatic photon itself to do work. Therefore, an infinite-staged Carnot heat engine model was established in our previous study [22] to investigate the spectral radiative exergy based on the concept of equivalent temperature and the differences between radiative energy and thermal energy. This theory represents the available energy of monochromatic radiation and corresponds to Petela’s equations macroscopically.

### 1.2. Second Law in Radiation Transfer Calculations

Lior et al. [26] reviewed the exergy calculations in the transfer process but radiation exergy was rarely considered in earlier studies. Caldas and Semiao [27] first considered the entropy generation in the radiation transfer calculations and analyzed the entropy generated by the emission, absorption, and scattering processes in the media independently. It was determined that the entropy calculation is entirely compatible with the typical radiative transfer calculation method. Later, the authors further discussed the effect of the interaction between turbulence and radiation on the entropy transfer in the media [28]. Liu and Chu [9] discussed the entropy generation of the radiation process and pointed out that it was a mistake to use the equation of conduction entropy generation to calculate the radiation transfer entropy generation. In addition, the calculation method of the radiative entropy generation proposed by Caldas and Semiao was verified using several examples. Subsequently, Liu and Chu [29] also extended this method to analyze the radiative entropy generation in enclosures filled with a semitransparent media and conducted verifications using two examples. Furthermore, Liu and Chu [30] developed a radiative exergy transfer equation based on the radiative exergy theory of Candau [23]. 

These studies on the theory of radiative exergy and the corresponding radiative exergy equations are based on the classical thermodynamic theory and they have focused on total spectral radiation engineering calculations such as the processes of flow, heat transfer, and combustion [31,32,33]. We do not disagree with these studies; however, they are not focused on analyzing the ability of monochromatic radiation to do work in spectral radiative transfer. In order to calculate the available energy of spectral radiation in a high-temperature energy conversion system, some problems need to be considered. First, the concept of radiation temperature [34] was used to represent the spectrum temperature; subsequently, the spectral radiative exergy was expressed. However, it is believed that the radiation temperature is a relatively macroscopic concept, which is not suitable to directly characterize the ability of the monochromatic radiation to do work. Second, both the spectral radiative entropy and the exergy in the radiation field have the same form of expression as the thermal energy, i.e., the exergy expression is similar to the Carnot efficiency expression. However, the thermal entropy or exergy form based on Carnot’s law is not suitable for characterizing the radiative entropy or the exergy. That is to say, it may be not appropriate to use the radiation temperature directly to calculate the spectral radiative exergy in the form of Carnot’s efficiency. Therefore, it is necessary to use a new spectral radiative exergy theory to perform a second law analysis in the spectral radiative transfer process based on the ability of the spectral radiation itself to do work; this is very important for flame energy quality-splitting conversion systems.

### 1.3. Summary

In order to better analyze the energy quality and its characteristics in the spectral radiation transfer for flame energy quality-splitting conversion systems, we use the more suitable monochromatic photon exergy theory [22] in the current research. The core ideas and equations of the monochromatic photon exergy theory presented in our previous study [22] are reviewed first based on the equivalent temperature and the infinite-staged Carnot model; subsequently, we provide a new discussion on this topic. Next, the spectral radiative entropy and exergy transfer equations are established based on the monochromatic photon exergy theory and both thermodynamic and numerical verifications are performed. Finally, some simplified one-dimensional furnace case calculations are provided to illustrate the application value of the new analysis method in engineering.

The main innovations and significance of this research include the following. (1) A new set of spectral radiative entropy and exergy transfer equations in participating media are established based on monochromatic photon exergy theory. (2) Spectral radiative exergy distribution characteristics are first studied and analyzed in one-dimensional furnace case. (3) Second law analytical and calculate methods are provided for the radiation transfer in flame energy gradient-utilization systems.

## 2. Spectral Radiative Exergy Theories Review and Comments 

Recently, we have established a monochromatic spectral radiative exergy theory [22] to characterize the ability to do work for monochromatic radiation; this is an extremely delicate model. The current study relies on this important idea to establish a spectral radiative exergy transfer equation; therefore, it is necessary to review and comment on this topic in this section.

### 2.1. Equivalent Temperature

It is considered that the monochromatic radiation is composed of many monochromatic photons; in the past, scholars have proposed a method to express photon exergy as photon energy multiplied by a Carnot efficiency coefficient [35,36] according to the second law of thermodynamics. However, the most representative concept is the equivalent temperature proposed by scholars such as Meszen et al. [37] and Chen et al. [24,25]. It is believed that the equivalent temperature should have the dimension of temperature and it is defined to express the quality of energy. 

### 2.2. Previous Research Review

**Figure 1:**
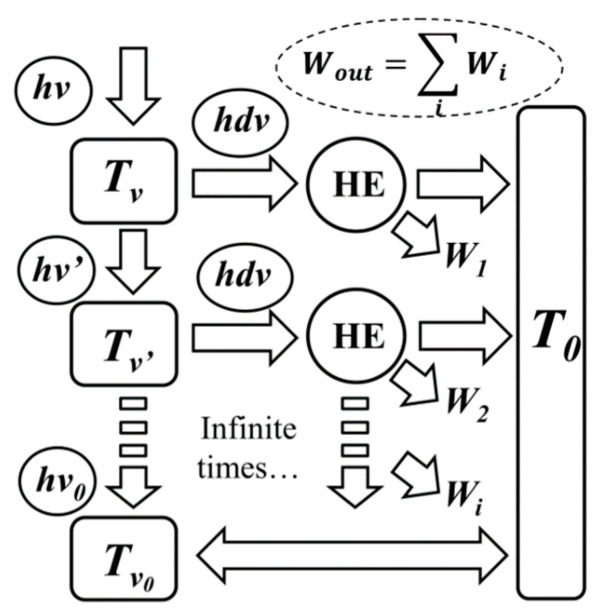
The infinite-staged Carnot engine model for the analysis of the monochromatic photon exergy [22].

Based on the concept of equivalent temperature and the differences between the radiative energy and thermal energy, an infinite-staged Carnot heat engine model was established to derive monochromatic photon exergy [22]. The model is shown in Figure 1, a radiation photon with a frequency *ν* and an equivalent temperature of *T_ν_* enters into an ideal absorption model, which emits a radiation photon with the frequency *ν*′ and energy *hν*′. Moreover, an infinitely small thermal energy *hdν* is generated at the same time and enters the Carnot heat engine with the equivalent temperature *T_ν_* and outputs work. After infinite times of such processes, the frequency drops to *ν*_0_ and the corresponding equivalent temperature becomes *T*_*v*0_, which is the same as the ambient temperature *T*_0_ to achieve equilibrium. It is appropriate to use the output work to represent the monochromatic photon exergy [22].

The monochromatic photon exergy derived from the above-mentioned processes can be expressed as an integral form [22]:

(1) $$E_{\nu }=\int_{\nu_{0}}^{\nu }h\left(1-\frac{T_{0}}{T_{\nu }}\right)d\nu .$$

It is considered that *T_ν_* in Equation (1) is related to the emission temperature and the frequency or the wavelength according to the relevant theory. If the equivalent temperature is expressed using the wavelength and Equation (1) is integrated to correspond to the macroscopic blackbody radiation exergy as described in Petela’s theory, the following equation is obtained [22]:

(2) $$T_{\lambda }^{4}=\frac{fT^{3}}{\lambda }.$$

Furthermore, the corresponding spectral radiative exergy–energy coefficient can be obtained using a comparison with the photon energy [22]:

(3) $$\eta_{\lambda }=\frac{E_{\nu }}{h\nu }=1-\frac{4}{3}\frac{T_{0}}{T_{\lambda }}+\frac{1}{3}{\left(\frac{T_{0}}{T_{\lambda }}\right)}^{4}.$$

This coefficient form is similar to that in Petela’s expression [6] for blackbody radiation exergy. Furthermore, based on actual engineering applications, an approximate equation was proposed to calculate the monochromatic photon equivalent temperature *T_λ_* from the radiation temperature *T* and wavelength *λ* [22]. When *T* > *T*_0_: $4.5638\times 10^{-3}T^{3}=\lambda T_{\lambda }^{4}$; when *T* < *T*_0_: $5.33\times 10^{-3}T^{3}=\lambda T_{\lambda }^{4}$.

In addition, the entropy of a monochromatic photon was also analyzed using the infinite-staged Carnot heat engine model and the entropy of the photon with frequency *ν* is [22]:

(4) $$S_{\nu }=\int_{0}^{\nu }\frac{hd\nu }{T_{\nu }}.$$

After substituting Equation (2) into Equation (4), the coefficient of the spectral radiative entropy to the energy can be obtained using a comparison with the photon energy [22]:

(5) $$\xi_{\nu }=\frac{S_{\nu }}{h\nu }=\frac{4}{3T_{\lambda }}.$$

### 2.3. Comments

Section 2.2 provides a brief review of the core ideas and main results of the monochromatic photon exergy theory [22]. Some new comments are provided in this section.

First, according to [22], the relationship between the monochromatic photon exergy and entropy represented by Equations (1) and (4) was proved to be in agreement with the Gouy–Stodola theorem:

(6) $$E=H-H_{0}-T_{0}\left(S-S_{0}\right).$$

Therefore, it indicates that the proposed entropy theory and exergy theory are intrinsically harmonious [22]. Moreover, the new spectral radiative exergy theory focuses on the ability of monochromatic radiation to do work and this ability is greater for shorter wavelength radiation. The radiative exergy in the whole blackbody spectrum corresponds to the blackbody radiative exergy expression proposed by Petela [10].

Second, it is evident that the equivalent temperature is explicitly defined to characterize the spectral radiative energy quality; this is different from the concept of radiation temperature, which is only related to the spectral radiation intensity. Moreover, the spectral radiative exergy coefficient derived from the infinite-staged Carnot heat engine model is different from the Carnot efficiency; this appropriately reflects the differences between the radiation and thermal energy. Therefore, the approach suggested in [22] seems to be more appropriate for the theoretical characterization of the spectral radiative exergy.

Finally, a special relationship between the monochromatic photons and spectral radiation is investigated from the perspective of radiative exergy. According to the infinite-staged Carnot engine model, for a photon of *hν*, *hν*_0_ is the reference state for calculating its radiative exergy. In addition, for black-body spectral radiation *I_b,λ_*, *I_b,λ_*(*T*_0_) is the reference state for calculating its radiation exergy [23,30]. It is believed that there should be a corresponding relationship between the macro and micro scales. Therefore, Equations (1) and (2) indicate the following relationship between the photons and the spectral radiation:

(7) $$\frac{I_{b,\lambda }\left(T_{0}\right)}{I_{b,\lambda }}=\frac{h\nu_{0}}{h\nu }=\frac{T_{0}^{4}}{T_{\lambda }^{4}}.$$

## 3. Thermodynamic Analysis of Spectral Radiation Transfer

### 3.1. Spectral Radiative Exergy Transfer

A spectral radiation transfer equation with emission, absorption, and scattering can be expressed as follows [1]:

(8) $$\frac{dI_{\lambda }\left(s\right)}{ds}=-\left(\kappa_{a,\lambda }+\kappa_{s,\lambda }\right)I_{\lambda }\left(s\right)+\kappa_{a,\lambda }I_{b,\lambda }+\frac{\kappa_{s,\lambda }}{4\pi }\int_{4\pi }I_{\lambda }\left(s{}^{\prime }\right)\Phi \left(s{}^{\prime },s\right)d\Omega {}^{\prime },$$

where *κ_a,λ_* is the spectral absorption coefficient; *κ_s,λ_* is the scattering coefficient, and Φ(**s**′,**s**) is the scattering phase function.

According to the spectral radiative exergy theory [22], the spectral radiative exergy transfer equation can be directly obtained:

(9) $$\frac{dE_{\lambda }(s)}{ds}=-\left(\kappa_{a,\lambda }+\kappa_{s,\lambda }\right)E_{\lambda }\left(s\right)+\kappa_{a,\lambda }I_{b,\lambda }\left(1-\frac{4}{3}\frac{T_{0}}{T_{\lambda }}+\frac{T_{0}^{4}}{3T_{\lambda }^{4}}\right)+\frac{\kappa_{s,\lambda }}{4\pi }\int_{4\pi }E_{\lambda }\left(s{}^{\prime }\right)\Phi \left(s{}^{\prime },s\right)d\Omega {}^{\prime }.$$

**Figure 2:**
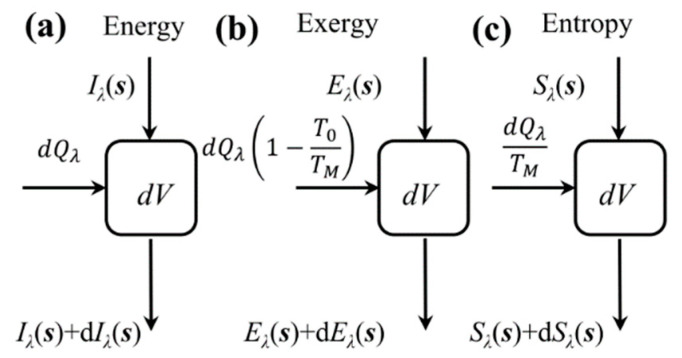
Radiation energy, exergy, and entropy transfer processes [29].

The first term on the right side of the equation represents the exergy intensity reduction due to absorption and scattering, the second term is the exergy of the emission, and the third term is the exergy enhancement in the direction of radiation transfer from the spatial scattering. Equation (9) reflects the spatial propagation process of the spectral radiative exergy. It can be seen in Figure 2 that the transfer process for the radiative exergy is very similar to the radiative energy transfer process. For radiation transfer in a participating media, the exergy is generated and it varies mainly in the radiation field, as well as in the media [30]. Therefore, the exergy loss in the participating media radiation transfer mainly includes the exergy loss in the radiation field and the exergy loss in the media because of radiation.

In order to determine the variation of the radiative exergy in the radiation field, the local net exergy loss of the radiation with a spectrum interval *dλ* after passing through the differential unit *dV* can be obtained by integrating Equation (9) over the entire solid angle of the space.

(10) $$\begin{array}{ll} dE_{R,\lambda }^{V} & =-\kappa_{a,\lambda }dVd\lambda \int_{4\pi }\left[E_{\lambda }\left(s\right)-I_{b,\lambda }\left(1-\frac{4}{3}\frac{T_{0}}{T_{\lambda }}+\frac{T_{0}^{4}}{3T_{\lambda }^{4}}\right)\right]d\Omega \\ & -\kappa_{s,\lambda }dVd\lambda \int_{4\pi }\left[E_{\lambda }\left(s\right)-\frac{1}{4\pi }\int_{4\pi }E_{\lambda }\left(s{}^{\prime }\right)\Phi \left(s{}^{\prime },s\right)d\Omega {}^{\prime }\right]d\Omega \end{array}.$$

Due to the radiation transfer in the participating media, the media also obtain or lose energy through radiation absorption or emission; thus, the exergy of the media varies [30]. As shown in Figure 2b, the local net exergy increment of the media due to the radiation heat flux can be calculated according to the definition of thermal exergy [30]:

(11) $$dE_{M,\lambda }^{V}=dQ_{\lambda }\left(1-\frac{T_{0}}{T_{M}}\right)=\kappa_{a,\lambda }dVd\lambda \left(1-\frac{T_{0}}{T_{M}}\right)\int_{4\pi }\left[I_{\lambda }\left(s\right)-I_{b,\lambda }\right]d\Omega .$$

Therefore, Equations (10) and (11) are combined. After the radiation of wavelength *λ* passes through a differential unit *dV* within the spectral interval *dλ*, the radiation exergy loss in the whole system is:

(12) $$\begin{array}{ll} dE_{RM,\lambda }^{V} & =-dE_{R,\lambda }^{V}-dE_{M,\lambda }^{V}\\ & =\kappa_{a,\lambda }dVd\lambda \int_{4\pi }\left[E_{\lambda }\left(s\right)-I_{\lambda }\left(s\right)\left(1-\frac{T_{0}}{T_{M}}\right)-I_{b,\lambda }\left(\frac{T_{0}}{T_{M}}-\frac{4}{3}\frac{T_{0}}{T_{\lambda }}+\frac{T_{0}^{4}}{3T_{\lambda }^{4}}\right)\right]d\Omega \\ & +\kappa_{s,\lambda }dVd\lambda \int_{4\pi }\left[E_{\lambda }\left(s\right)-\frac{1}{4\pi }\int_{4\pi }E_{\lambda }\left(s{}^{\prime }\right)\Phi \left(s{}^{\prime },s\right)d\Omega {}^{\prime }\right]d\Omega \end{array}.$$

Because $I_{b,\lambda }\frac{T_{0}^{4}}{3T_{\lambda }^{4}}=\frac{1}{3}I_{b,\lambda }\left(T_{0}\right)$, according to Equation (7), which is constant for a radiation with wavelength *λ* in a spectrum of *dλ*; that is, its value does not change after passing through a differential unit *dV*; therefore:

(13) $$dVd\lambda \int_{4\pi }I_{b,\lambda }\frac{T_{0}^{4}}{3T_{\lambda }^{4}}d\Omega =dVd\lambda \int_{4\pi }\frac{1}{3}I_{b,\lambda }\left(T_{0}\right)d\Omega =0.$$

Therefore, Equation (12) is rewritten as:

(14) $$\begin{array}{ll} dE_{RM,\lambda }^{V} & =-dE_{R,\lambda }^{V}-dE_{M,\lambda }^{V}\\ & =\kappa_{a,\lambda }dVd\lambda \int_{4\pi }\left[E_{\lambda }\left(s\right)-I_{\lambda }\left(s\right)\left(1-\frac{T_{0}}{T_{M}}\right)-I_{b,\lambda }\left(\frac{T_{0}}{T_{M}}-\frac{4}{3}\frac{T_{0}}{T_{\lambda }}\right)\right]d\Omega \\ & +\kappa_{s,\lambda }dVd\lambda \int_{4\pi }\left[E_{\lambda }\left(s\right)-\frac{1}{4\pi }\int_{4\pi }E_{\lambda }\left(s{}^{\prime }\right)\Phi \left(s{}^{\prime },s\right)d\Omega {}^{\prime }\right]d\Omega \end{array}.$$

The first term on the right side of the equation is the radiative exergy loss caused by the absorption and emission process and the second term is the exergy loss caused by the scattering process.

### 3.2. Spectral Radiative Entropy Transfer

According to the monochromatic photon entropy expression described in Section 2, a spectral radiative entropy transfer equation can be derived by analogy with the radiative exergy transfer Equation (9):

(15) $$\frac{dS_{\lambda }\left(s\right)}{ds}=-\left(\kappa_{a,\lambda }+\kappa_{s,\lambda }\right)S_{\lambda }\left(s\right)+\kappa_{a,\lambda }\frac{4I_{b,\lambda }}{3T_{\lambda }}+\frac{\kappa_{s,\lambda }}{4\pi }\int_{4\pi }S_{\lambda }\left(s{}^{\prime }\right)\Phi \left(s{}^{\prime },s\right)d\Omega {}^{\prime }.$$

The first term on the right side of the equation represents the entropy intensity reduction due to absorption and scattering, the second term is the entropy of the emission, and the third term is the entropy enhancement in the direction of radiation transfer from the spatial scattering. Equation (15) reflects the spatial transfer process of radiative entropy. It can be seen in Figure 2 that the transfer process for the radiative entropy is very similar to the radiative energy and exergy transfer processes. Therefore, the entropy generation in the participating media radiation transfer also includes the entropy generation in the radiation field and the entropy generation in the medium caused by the radiation [27,29].

For the radiative entropy generation in the radiation field, the local net entropy increment of the radiation with the spectrum interval *dλ* after passing through the differential unit *dV* can be obtained by integrating Equation (15) over the entire solid angle of the space:

(16) $$\begin{array}{ll} dS_{R,\lambda }^{V} & =-\kappa_{a,\lambda }dVd\lambda \int_{4\pi }\left[S_{\lambda }\left(s\right)-\frac{4I_{b,\lambda }}{3T_{\lambda }}\right]d\Omega \\ & -\kappa_{s,\lambda }dVd\lambda \int_{4\pi }\left[S_{\lambda }\left(s\right)-\frac{1}{4\pi }\int_{4\pi }S_{\lambda }\left(s{}^{\prime }\right)\Phi \left(s{}^{\prime },s\right)d\Omega {}^{\prime }\right]d\Omega \end{array}.$$

Due to the radiation transfer in the participating media, the media also obtain or lose energy through radiation absorption or emission; thus, the entropy of the media varies [27,29]. As shown in Figure 2c, the local net entropy increment of the media due to the radiation heat flux can be calculated according to the definition of thermal entropy [27,29]:

(17) $$dS_{M,\lambda }^{V}=\frac{dQ_{\lambda }}{T_{M}}=\kappa_{a,\lambda }dVd\lambda \int_{4\pi }\frac{I_{\lambda }\left(s\right)-I_{b,\lambda }}{T_{M}}d\Omega .$$

Therefore, Equations (16) and (17) are combined. After the radiation of wavelength *λ* passes through a differential unit *dV* within the spectral interval *dλ*, the radiative entropy generation in the whole system is:

(18) $$\begin{array}{ll} dS_{RM,\lambda }^{V} & =dS_{R,\lambda }^{V}+dS_{M,\lambda }^{V}\\ & =-\kappa_{a,\lambda }dVd\lambda \int_{4\pi }\left[S_{\lambda }\left(s\right)-\frac{4I_{b,\lambda }}{3T_{\lambda }}-\frac{I_{\lambda }\left(s\right)}{T_{M}}+\frac{I_{b,\lambda }}{T_{M}}\right]d\Omega \\ & -\kappa_{s,\lambda }dVd\lambda \int_{4\pi }\left[S_{\lambda }\left(s\right)-\frac{1}{4\pi }\int_{4\pi }S_{\lambda }\left(s{}^{\prime }\right)\Phi \left(s{}^{\prime },s\right)d\Omega {}^{\prime }\right]d\Omega \end{array}.$$

The first term on the right side of the equation is the radiative entropy generation caused by the absorption and emission processes and the second term is the entropy generation caused by the scattering process.

The spectral radiative exergy transfer equation (Equation (9)), the entropy transfer equation (Equation (15)), and the exergy loss or entropy generation in the radiation transfer process can be combined with various radiation transfer numerical calculation methods, such as the discrete ordinate method (DOM), finite volume method (FVM), discrete transfer method, and spherical harmonics method [1]. In this study, some examples are analyzed using the widely applicable DOM.

### 3.3. Verification

#### 3.3.1. Thermodynamic Relationship Verification

According to the Gouy–Stodola theorem in Equation (6), the relationship between the radiative entropy and exergy could be verified.

First, for the spectral radiative entropy transfer equation and exergy transfer equation, according to Gouy–Stodola theorem, there is:

(19) $$E_{\lambda }=I_{\lambda }-I_{b,\lambda }\left(T_{0}\right)-T_{0}\left[S_{\lambda }-S_{b,\lambda }\left(T_{0}\right)\right],$$

and:

(20) $$\frac{dE_{\lambda }}{ds}=\frac{d\left[I_{\lambda }-I_{b,\lambda }\left(T_{0}\right)\right]}{ds}-T_{0}\frac{d\left[S_{\lambda }-S_{b,\lambda }\left(T_{0}\right)\right]}{ds}.$$

After substituting the radiation transfer equation (Equation (8)) and the spectral radiative entropy transfer equation (Equation (15)) into the right side of the Equation (20), the following is obtained:

(21) $$\begin{array}{l} \;\frac{d\left[I_{\lambda }-I_{b,\lambda }\left(T_{0}\right)\right]}{ds}-T_{0}\frac{d\left[S_{\lambda }-S_{b,\lambda }\left(T_{0}\right)\right]}{ds}\\ =-\left(\kappa_{a,\lambda }+\kappa_{s,\lambda }\right)\left\{I_{\lambda }\left(s\right)-I_{b,\lambda }\left(T_{0}\right)-T_{0}\left[S_{\lambda }\left(s\right)-S_{b,\lambda }\left(T_{0}\right)\right]\right\}\\ \;+\kappa_{a,\lambda }\left\{I_{b,\lambda }-I_{b,\lambda }\left(T_{0}\right)-T_{0}\left[\frac{4I_{b,\lambda }}{3T_{\lambda }}-\frac{4I_{b,\lambda }\left(T_{0}\right)}{3T_{0}}\right]\right\}\\ \;+\frac{\kappa_{s,\lambda }}{4\pi }\int_{4\pi }\left\{I_{\lambda }\left(s{}^{\prime }\right)-I_{b,\lambda }\left(T_{0}\right)-T_{0}\left[S_{\lambda }\left(s{}^{\prime }\right)-S_{b,\lambda }\left(T_{0}\right)\right]\right\}\Phi \left(s{}^{\prime },s\right)d\Omega {}^{\prime } \end{array}.$$

Consider the second item on the right side of Equation (21):

(22) $$I_{b,\lambda }-I_{b,\lambda }\left(T_{0}\right)-T_{0}\left[\frac{4I_{b,\lambda }}{3T_{\lambda }}-\frac{4I_{b,\lambda }\left(T_{0}\right)}{3T_{0}}\right]=I_{b,\lambda }\left[1-\frac{4T_{0}}{3T_{\lambda }}+\frac{I_{b,\lambda }\left(T_{0}\right)}{3I_{b,\lambda }}\right].$$

According to Equation (7), there is:

(23) $$I_{b,\lambda }\left[1-\frac{4T_{0}}{3T_{\lambda }}+\frac{I_{b,\lambda }\left(T_{0}\right)}{3I_{b,\lambda }}\right]=I_{b,\lambda }\left(1-\frac{4T_{0}}{3T_{\lambda }}+\frac{T_{0}^{4}}{3T_{\lambda }^{4}}\right).$$

Therefore, Equation (21) could be written as:

(24) $$\begin{array}{l} \;\frac{d\left[I_{\lambda }-I_{b,\lambda }\left(T_{0}\right)\right]}{ds}-T_{0}\frac{d\left[S_{\lambda }-S_{b,\lambda }\left(T_{0}\right)\right]}{ds}\\ =-\left(\kappa_{a,\lambda }+\kappa_{s,\lambda }\right)E_{\lambda }\left(s\right)+\kappa_{a,\lambda }I_{b,\lambda }\left(1-\frac{4T_{0}}{3T_{\lambda }}+\frac{T_{0}^{4}}{3T_{\lambda }^{4}}\right)\\ \;+\frac{\kappa_{s,\lambda }}{4\pi }\int_{4\pi }E_{\lambda }\left(s{}^{\prime }\right)\Phi \left(s{}^{\prime },s\right)d\Omega {}^{\prime }\\ =\frac{dE\left(s\right)}{ds} \end{array}.$$

This corresponds to the Gouy–Stodola theorem defined in Equation (19).

The relationship between the spectral radiative entropy generation and exergy loss according to the Gouy–Stodola theorem is:

(25) $$dE_{RM,\lambda }^{V}=dI_{R,\lambda }^{V}+dQ_{M,\lambda }^{V}-T_{0}dS_{RM,\lambda }^{V}.$$

Using Equation (8) for the radiative intensity and Equation (18) for entropy and considering Equation (7), the following is obtained:

(26) $$\begin{array}{l} \;dI_{R,\lambda }^{V}+dQ_{M,\lambda }^{V}-T_{0}dS_{RM,\lambda }^{V}\\ =-\kappa_{a,\lambda }dVd\lambda \int_{4\pi }\left[I_{\lambda }\left(s\right)-I_{b,\lambda }-I_{\lambda }\left(s\right)+I_{b,\lambda }\right]d\Omega \\ \;-\kappa_{s,\lambda }dVd\lambda \int_{4\pi }\left[I_{\lambda }\left(s\right)-\frac{1}{4\pi }\int_{4\pi }I_{\lambda }\left(s{}^{\prime }\right)\Phi \left(s{}^{\prime },s\right)d\Omega {}^{\prime }\right]d\Omega \\ \;-\kappa_{a,\lambda }dVd\lambda \int_{4\pi }\left[T_{0}S_{\lambda }\left(s\right)-I_{b,\lambda }\frac{4T_{0}}{3T_{\lambda }}-I_{\lambda }\left(s\right)\frac{T_{0}}{T_{M}}+I_{b,\lambda }\frac{T_{0}}{T_{M}}\right]d\Omega \\ \;-\kappa_{s,\lambda }dVd\lambda \int_{4\pi }\left[T_{0}S_{\lambda }\left(s\right)-\frac{1}{4\pi }\int_{4\pi }T_{0}S_{\lambda }\left(s{}^{\prime }\right)\Phi \left(s{}^{\prime },s\right)d\Omega {}^{\prime }\right]d\Omega \\ =\kappa_{a,\lambda }dVd\lambda \int_{4\pi }\left[E_{\lambda }\left(s\right)-I_{\lambda }\left(s\right)\left(1-\frac{T_{0}}{T_{M}}\right)-I_{b,\lambda }\left(\frac{T_{0}}{T_{M}}-\frac{4}{3}\frac{T_{0}}{T_{\lambda }}\right)\right]d\Omega \\ \;+\kappa_{s,\lambda }dVd\lambda \int_{4\pi }\left[E_{\lambda }\left(s\right)-\frac{1}{4\pi }\int_{4\pi }E_{\lambda }\left(s{}^{\prime }\right)\Phi \left(s{}^{\prime },s\right)d\Omega {}^{\prime }\right]d\Omega \\ =dE_{RM,\lambda }^{V} \end{array}.$$

It can be seen that the entropy generation and exergy loss in the radiation process agree with the laws of thermodynamics, demonstrating that the entropy generation and the exergy loss are uniform and in internal harmony in the radiation transfer process.

#### 3.3.2. Numerical Verification

Here we reference the numerical verification method proposed by Liu and Chu [29] to verify the new radiative entropy transfer equation. A one-dimensional case is used with equally high temperatures of the two boundary walls; the wall temperatures are set to 3500 K, 2500 K, and 1750 K. The temperature of the absorbing and emission media is constant at 350 K, the absorption coefficient is set to 0.5, and the length is 1 m. The DOM is used for the calculation and the discrete format is S_8_. This method has been applied in the calculation of the one-dimensional radiative heat flux. [38,39]

By integrating Equation (18) on a one-dimensional space and the whole spectrum, we can obtain the entropy generation of the radiation transfer in a one-dimensional space [29]:

(27) $$\Gamma_{G}=\int_{V}\int_{\lambda }dS_{RM,\lambda }^{V}d\lambda dV.$$

The dimensionless entropy generation in a one-dimensional space is defined as [29]:

(28) $$\Pi_{G}=\frac{\Gamma_{G}T_{L}}{Q}.$$

**Figure 3:**
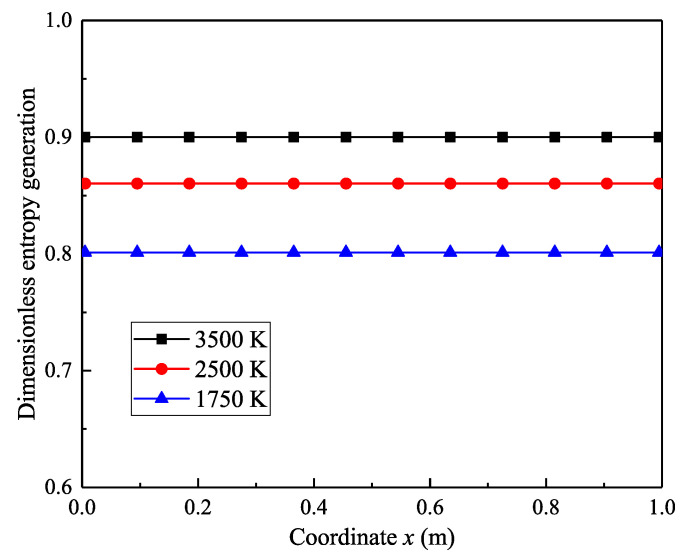
Numerical verification results of dimensionless radiative entropy generation in the one-dimensional case.

The numerical results of the dimensionless entropy generation in the one-dimensional space are shown in Figure 3. It can be seen from Figure 3 that the dimensionless entropy production at different coordinates in the one-dimensional space was stable at 0.9, 0.86, and 0.8.

On the other hand, as demonstrated in [29], the whole system does not output work; therefore, the entropy generation can be predicted by the theoretical method:

(29) $$\Pi_{G}=\left(\frac{Q}{T_{L}}-\frac{Q}{T_{H}}\right)\frac{T_{L}}{Q}=1-\frac{T_{L}}{T_{H}}.$$

The results of the theoretical calculation (Equation (29)) indicate that the dimensionless entropy generation in the one-dimensional space is 0.9, 0.86, and 0.8 for the wall temperatures of 3500 K, 2500 K, and 1750 K, respectively. A comparison between theoretical results and numerical results in Figure 3 shows that the numerical entropy production in the whole space is very similar to the theoretical results. According to [29], this approach verifies the new spectral radiative exergy theory and numerical calculation method of the second law calculation for the spectral radiation transfer.

## 4. One-Dimensional Cases Studies

### 4.1. Cases and Calculation Method

#### 4.1.1. One-Dimensional Cases

In this study, one-dimensional infinite large parallel plates are selected to represent different high-temperature combustion engineering applications.

(1) Case 1 is a one-dimensional furnace temperature field, representing a gas-burning furnace. The one-dimensional length *L* is 1 m and is divided into 100 grids. The plate is a black body wall with a temperature of 1000 K; the one-dimensional temperature distribution is [38]:

(30) $$T=1400-400\cos\left(\frac{2\pi x}{L}\right)\left(K\right).$$

**Figure 4:**
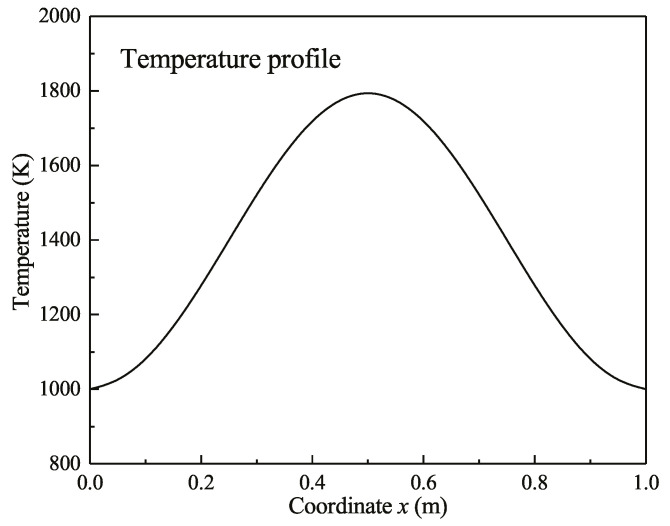
Temperature profile of the one-dimensional case.

The temperature distribution is shown in Figure 4; the absorption coefficient $\kappa_{a}$ is 0.43 [31] and ambient temperature *T*_0_ is 300 K for the exergy calculation.

(2) Case 2 is a one-dimensional scattering furnace temperature field that represents a heavy oil- or coal-burning furnace. The temperature field and absorption coefficient are the same as in case 1. In [27], the scattering coefficient *κ*_s_ is in the range of 0.01–0.2 to model a two-dimensional furnace; thus, the *κ*_s_ is simplified as 0.1 in the current study. Moreover, the scattering phase function is set as $\Phi \left(\theta_{i},\theta_{s}\right)=1+0.6\cos\theta_{i}\cos\theta_{s}$ [27], where *θ* is the zenith angle of incident direction or scattering direction.

#### 4.1.2. Calculation Method

The DOM is used for the radiative exergy transfer calculation. The S_8_ discrete format is used for the one-dimensional case, the detail of this method is introduced in [1]. The radiative exergy represented by the transfer Equation (9) is discretely solved at each grid point. The specific process is same as the solution of the one-dimensional radiation transfer equation in our latest published research [38,39] and the procedure is written by Fortran code. The radiation exergy flux in the radiation field is calculated according to the radiative exergy transfer equation:

(31) $$e=\int_{4\pi }\int_{0}^{\infty }\left(E_{\lambda }^{+}\left(s\right)-E_{\lambda }^{-}\left(s\right)\right)d\lambda d\Omega .$$

The ‘+’ and ‘−’ symbols indicate the two directions of transmission, i.e., right and left, respectively; this is similar to the calculation of the radiative heat flux [38,39].

According to the black body radiation distribution, most of the radiation occurs in the near-infrared region in the high-temperature furnace and the radiation intensity is weak for the wavelength above 50 μm. Therefore, the radiation intensity in the range of 0–50 μm wavelength is used to represent the entire radiation intensity in the calculation. The wavelength interval is 25 nm. The spectral radiative exergy distribution in the 0–50 μm wavelength range is investigated according to the distribution of the spectral radiation intensity of natural gas combustion [40,41].

### 4.2. Calculation Results

#### 4.2.1. Results of the Temperature Field without Scattering

**Figure 5:**
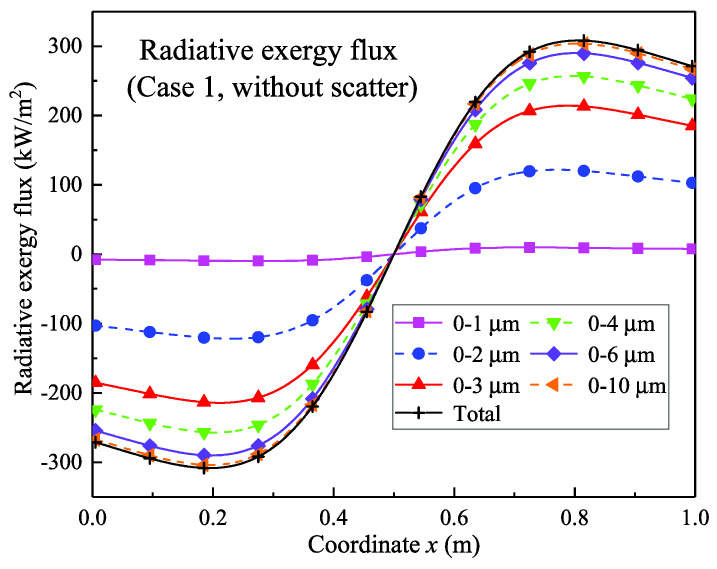
One-dimensional distribution of the radiation exergy flux in non-scattering conditions.

**Figure 6:**
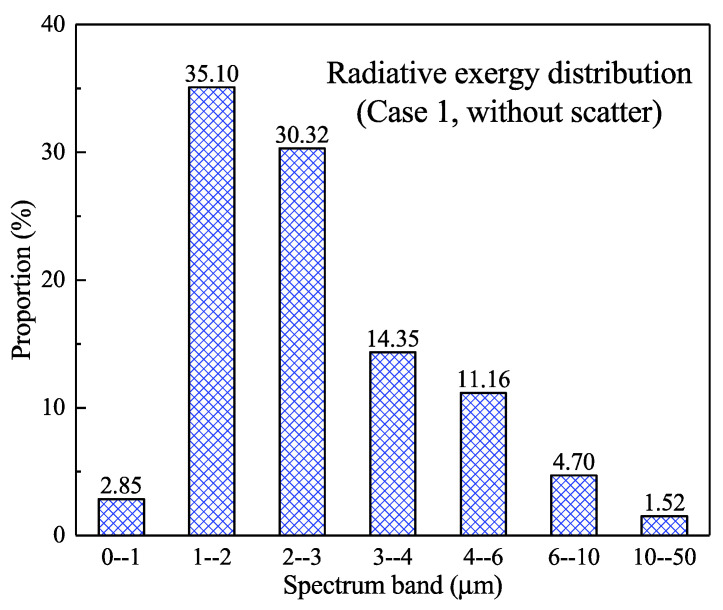
The proportion of the radiative exergy flux in different spectral bands near the wall (non-scattering condition).

**Figure 7:**
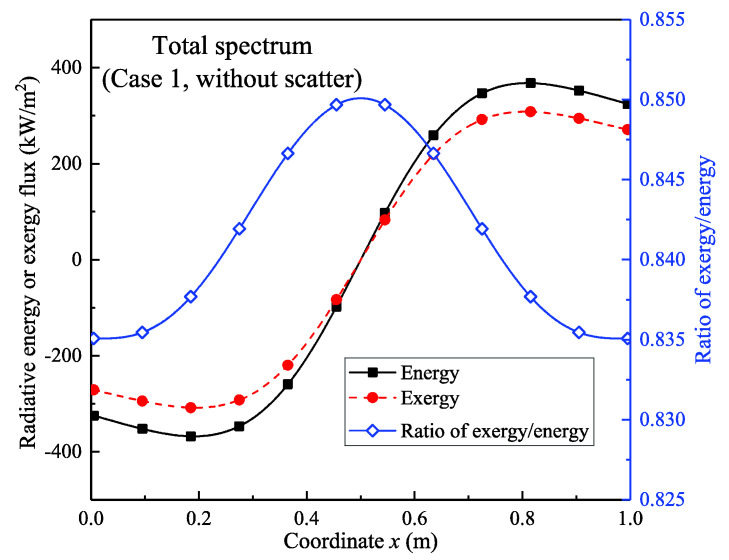
Radiative energy and exergy flux distribution and energy–exergy ratio (0–50 μm; non-scattering condition).

The non-scattering results are shown in Figure 5, Figure 6 and Figure 7. Moreover, Figure 5 shows the radiative exergy flux at each point in the transfer process; the positive values represent the rightward transmission and the negative values represent the leftward transmission. It can be seen that the radiative exergy flux at the midpoint was 0 in Figure 5 and the exergy flux increased in the left and right directions. The temperature distribution (Figure 4) shows that the radiative exergy flux in the high-temperature furnace is transferred from the high-temperature flue gas media to the furnace wall surfaces on each side; this is the same direction as that of the radiative heat flux [39]. The maximum radiative exergy flux of 308.3 kW/m^2^ occurred at the 1/4 width point from the left or right wall. It occurs at this location because this is where the temperature change is largest.

In addition, Figure 5 also shows the radiative exergy flux in the different spectral bands. The exergy flux in the 0–1 μm band is small and its maximum value was 9.78 kW/m^2^. The maximum radiative exergy flux in the 0–2 μm band reached 121.84 kW/m^2^, whereas the value increased to 214.3 kW/m^2^ in the 0–3 μm band. The exergy flux continues to increase as the spectral range increases but the rate of increase decreases significantly. Figure 6 further shows the proportional distribution of the radiative exergy flux in the different spectral bands near the wall. It can be seen that the radiative exergy flux in the 0–1 μm band was relatively small with a proportion of 2.85%, whereas the 1–2 μm band had the largest proportion of 35.1%. Furthermore, the 2–3 μm band represented 30.32%, which ranks second after the 1–2 μm band. After that, the proportion gradually decreased and the bands above 10 μm had a proportion of only 1.52%. In general engineering application, the bands below 1 μm are in the visible light range and directly stimulate the photoelectric effect of silicon cells; moreover, visible light promotes photosynthesis and some photochemical reactions. However, the results show that the exergy flux of this band is low in the high-temperature combustion. It can also be seen from Figure 6 that the radiative exergy flux in the 1–3 μm band accounted for more than 60%; therefore, the effective utilization of this band will directly improve the conversion efficiency of the combustion radiation exergy. For a combustion flame grading power generation system, the infrared radiation below 3 μm could be used for photovoltaic conversion and it matches the thermo-photovoltaic cells of materials such as GaSb, InGaSb, and InGaAsSb, but these materials are costly or are still in the research stage. For infrared radiation above 3 μm, thermal utilization seems to be a suitable conversion route, this corresponds to the thermal power cycle conversion.

Figure 7 shows the 0–50 μm full-spectrum band radiative exergy flux versus the radiative energy flux and the exergy-energy ratio is also investigated. The results indicate that the difference between the energy flux and the exergy flux is small in the central section and the difference increases toward the walls. This occurs mainly because the absolute values of the energy flux and the exergy flux increase toward the walls. Moreover, the exergy–energy ratio was approximately 0.85 near the midpoint and it gradually decreased toward both walls; the ratio near the two walls was 0.835. This occurs mainly because the intermediate temperature is high and the temperatures near the walls are low. According to Equations (2) and (3), the exergy–energy ratio is related to the temperature. In general, the radiative exergy–energy ratio is greater at a higher temperature.

#### 4.2.2. Results of the Temperature Field with Scattering

**Figure 8:**
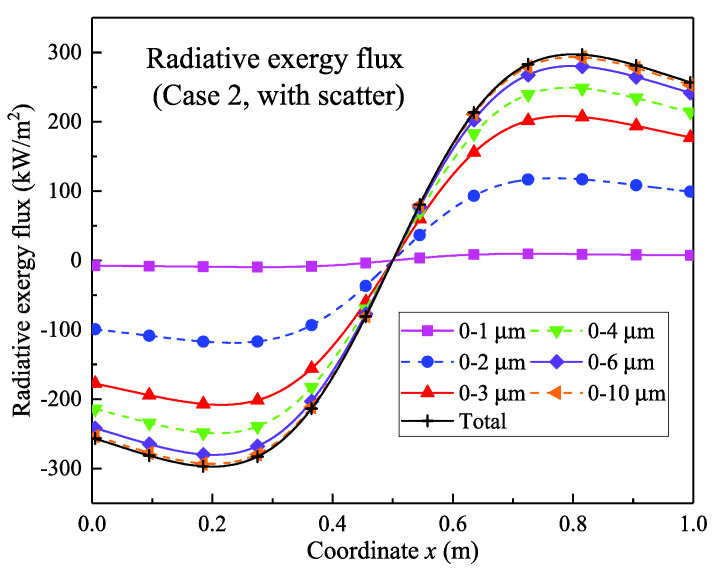
One-dimensional distribution of the radiative exergy flux in the scattering condition.

**Figure 9:**
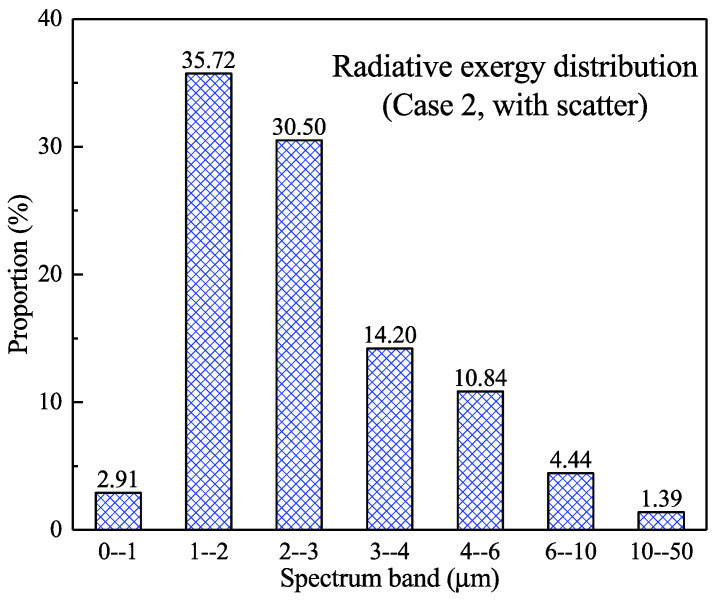
The proportion of the radiative exergy flux in different spectral bands near the wall (scattering condition).

Figure 8 and Figure 9 show the calculation results of the radiative exergy flux of the scattering case 2. It can be seen from Figure 8 that the results were similar to those of the non-scattering case; the radiative exergy flux was propagated from the center to the two walls. The radiative exergy flux in the different bands was similar to that of the non-scattering case. The maximum radiative exergy flux was 297.4 kW/m^2^, which was slightly smaller than the value of 308.3 kW/m^2^ for the non-scattering case. In addition, the exergy flux in the different spectral bands was also lower. This indicates that the scattering condition weakens the radiation transfer intensity, thereby reducing the exergy flux.

Figure 9 further shows the proportional distribution of the radiative exergy flux in different spectral bands near the wall under the scattering condition. The results were similar to those of the no-scattering condition shown in Figure 6. The exergy flux in the 0–1 μm band accounted for only 2.91%, whereas the largest proportion of 35.72% occurred in the 1–2 μm band; moreover, the exergy flux in the 2–3 μm band represented 30.50%. It should be noted that the radiative exergy flux in the 0–3 μm band constituted 0.86% higher than that in the non-scattering condition, whereas the spectral radiative exergy above 3 μm was slightly less than that in the non-scattering condition. 

**Figure 10:**
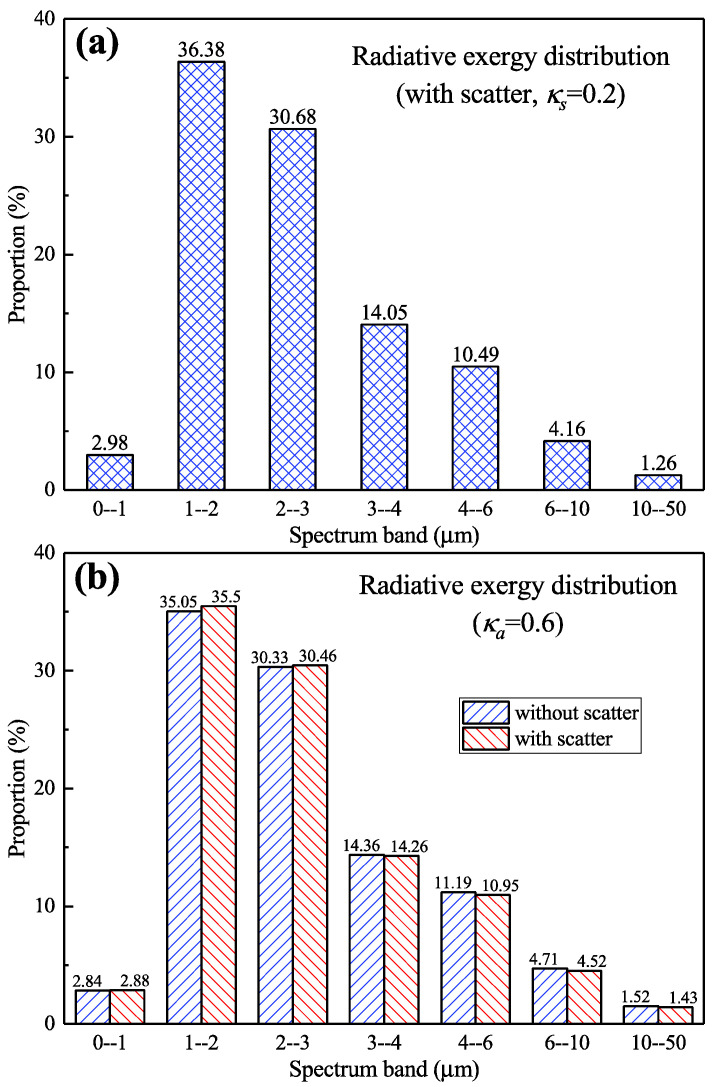
The proportion of the radiative exergy flux in different spectral bands near the wall. (**a**) Different scattering coefficient; (**b**) different absorption coefficient.

It can be seen that the scattering improves the proportion of the short wavelength radiative exergy in the radiation transfer process (Figure 9). Furthermore, we changed the scattering and absorbing coefficient to investigate this phenomenon and the results are shown in Figure 10. It can be seen in Figure 10a that after the scattering coefficient becoming 0.2, the radiative exergy flux in the 0–3 μm band is 1.77% higher than that in the non-scattering condition (case 1 in Figure 6). Figure 10b shows that the radiative exergy flux in the 0–3 μm band with scattering is 0.62% higher than that without scattering after the absorption coefficient becoming 0.6. In addition, we also found that the exergy flux was lower in the scattering condition than the non-scattering condition after the scattering and absorption coefficients had changed. Therefore, these results imply that scattering could slightly improve the proportion of radiative exergy below 3 μm in the radiation transfer process. This indicates that the use of solid fuel in the combustion chamber might improve the proportion of the short wavelength radiative exergy, thereby providing guidance for improving the combustion flame grading power generation system. However, since the proportion improvement is small and the model is relatively simplified, further experiments and detailed numerical simulation analysis are needed in the future.

**Figure 11:**
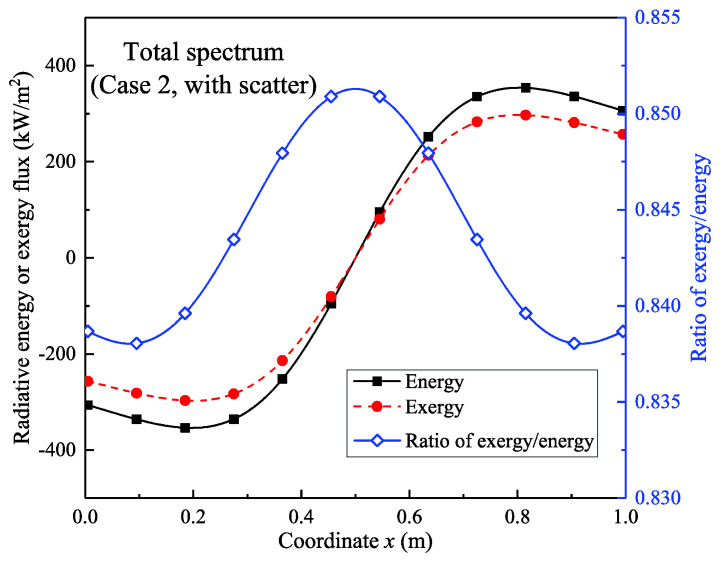
Radiative energy and exergy flux distribution and exergy–energy ratio (0–50 μm; scattering condition).

Figure 11 shows the total radiative energy and exergy flux in the spectral range of 0–50 μm under scattering conditions (case 2); the exergy–energy ratio is also illustrated. The exergy–energy ratio was approximately 0.851 near the midpoint and the ratio was lower towards the walls; thus, there was a minimum ratio of 0.838 near the wall surface. A comparison with the non-scattering results (Figure 7) shows that, when scattering occurred, the radiative exergy to energy ratio was about 0.003 higher than without scattering. This indicates that scattering might have the effect to improve the exergy–energy ratio; however, this is a very small improvement, further research is also needed to verify this effect.

The results for the one-dimensional furnace cases show that the radiative exergy flux in the radiation transfer process can be numerically calculated using Equation (9) and combining the second law and the radiation transfer process. Furthermore, the influences of different conditions on the energy and exergy distribution in the radiation transfer process can be investigated, which has practical application value for the combustion flame grading power generation system.

## 5. Conclusions

By considering the second law of thermodynamics, this study combines the radiation transfer equation with the theory of the monochromatic photon exergy developed in previous research [18]. The spectral radiative entropy transfer equation and radiative exergy transfer equation are established based on the second law; this process can be used to numerically analyze the variability of the energy quality more appropriately and accurately during the spectral radiation transfer process. It is found that the radiative entropy and the radiative exergy are consistent with the thermodynamic Gouy–Stodola law. 

The distribution and variation of the radiation spectral exergy flux during the radiation transfer process can be obtained using numerical calculations of the spectral radiative exergy transfer equation. It is also possible to calculate the distribution of the spectral radiative exergy, which is of great significance for the analysis of the flame energy spectrum-splitting conversion system in high-temperature engineering. The calculation results of one-dimensional furnace cases show that the radiative exergy in the 1–3 μm band accounts for the largest proportion. The presence of scattering reduces the radiative exergy flux but could slightly increase the proportion of the radiative exergy flux at short wavelengths below 3 μm to some extent.

## Nomenclature

*e*	radiative exergy flux
*E*	radiative exergy intensity
*E_v_*	exergy of monochromatic photon
*f*	coefficient of equivalent temperature
*h*	Planck constant
*H*	enthalpy
*I*	radiative intensity
*L*	distance between one dimensional parallel infinite plates
*Q*	thermal energy
*s*	direction of radiation transfer
*s*′	direction of incident scattering radiation
*S*	radiative entropy intensity
*S_v_*	entropy of monochromatic photon
*T*	temperature
*T*_0_	ambient temperature
*V*	volume
*x*	length coordinate
*Geek symbols*	
*Γ_G_*	radiative entropy generation in one-dimensional space
*η*	exergy-to-energy coefficient
*θ*	zenith angle
*κ*	absorption coefficient
*λ*	Wavelength
*ν*	Frequency
*ξ*	entropy-to-energy coefficient
*Π_G_*	dimensionless radiative entropy generation in one-dimensional space
*Φ*	scattering phase function
*Ω*	solid angle
*Ω*′	solid angle of incident scattering
*Subscripts*	
*a*	absorption
*b*	blackbody
*H*	high
*L*	low
*M*	media
*R*	radiation field
*s*	scattering
*0*	reference state
